# Ubiquitin-like and ubiquitinylated proteins associated with the maternal cell walls of *Scenedesmus obliquus* 633 as identified by immunochemistry and LC–MS/MS proteomics

**DOI:** 10.1007/s00709-024-01994-3

**Published:** 2024-10-04

**Authors:** Justyna Kowalczyk, Kinga Kłodawska, Maria Zych, Jan Burczyk, Przemysław Malec

**Affiliations:** 1 Department of Plant Physiology and Biochemistry, Faculty of Biochemistry, Biophysics and Biotechnology, Jagiellonian University, 30-387 Kraków, Poland; 2 Doctoral School of Exact and Natural Sciences, Jagiellonian University, 30-348 Kraków, Poland; 3 Department of Pharmacognosy and Phytochemistry, Faculty of Pharmaceutical Sciences in Sosnowiec, Medical University of Silesia, Katowice, Jagiellońska 4, 41-200 Sosnowiec, Poland; 4 Laboratory of Biotechnology, Puńcowska 74, 43-400 Cieszyn, Poland

**Keywords:** *Scenedesmus*, Extramembranous compartment, Cell walls, Proteome, Ubiquitin

## Abstract

The cell walls of green algae *Scenedesmus obliquus* are complex, polymeric structures including an inner cellulose layer surrounded by an algaenan-containing trilaminar sheath. The process of autosporulation leads to the formation of sporangial (maternal) cell walls, which are released into the medium after sporangial autolysis. In this study, a fraction of maternal cell wall material (CWM) was isolated from the stationary phase cultures of *Scenedesmus obliquus* 633 and subjected to immunofluorescence microscopy using polyclonal anti-ubiquitin antibodies. The water-extracted polypeptide fraction from the maternal cell walls was then analyzed using immunoblotting and LC–MS/MS. An immunoanalysis showed the presence of several peptides reactive with polyclonal anti-ubiquitin serum, with apparent molecular masses of c. 12, 70, 120, 200, and > 250 kDa. Cell wall–associated peptides were identified on the basis of LC–MS/MS spectra across NCBI databases, including the *Scenedesmaceae* family (58 records), the *Chlorophyceae* class (37 records), and *Chlamydomonas reinhardtii* (18 records) corresponding to the signatures of 95 identified proteins. In particular, three signatures identified ubiquitin and ubiquitin-related proteins. In the maternal cell walls, immunoblotting analysis, immunofluorescence microscopy, and LC–MS/MS proteomics collectively demonstrated the presence of ubiquitin-like epitopes, ubiquitin-specific peptide signatures, and several putative ubiquitin conjugates of a higher molecular mass. These results support the presence of ubiquitin-like proteins in the extramembranous compartment of *Scenedesmus obliquus* 633 and suggest that protein ubiquitination plays a significant role in the formation and functional integrity of the maternal cell walls in green algae.

**Supplementary Information:**

The online version contains supplementary material available at 10.1007/s00709-024-01994-3.

## Introduction

*Chlorophyta* are one of the most diverse groups of eukaryotic algae, with increasing potential for commercial applications (Aarthy et al. 2018; Pan et al. 2019; Skjånes et al. 2013; Spain et al. 2021; Spolaore et al. 2006; Wang et al. 2021). In particular, algae from the genus *Scenedesmus*, the cosmopolitically distributed freshwater *Chlorophyta* (Lürling 2003), have attracted global attention (Ishaq et al. 2016; Mandal and Mallick 2009).

In algae, the cell walls are extramembranous structures surrounding the cell that enable interactions with the environment and provide protection against different types of stressors (Domozych 2019). Due to their high mechanical and chemical stability, cell walls are often a serious obstacle in the acquisition of biotechnologically useful algal metabolites (Ventura et al. 2017).

The cell walls of *S. obliquus* consist of two layers: the inner microfibrillar layer is surrounded by a trilaminar sheath (see: Baudelet et al. 2017, for a review). The surface of the cell walls in *Scenedesmus* spp. is frequently covered with various epistructures that are characteristic of strains. It has been postulated that some of them may contribute to the lower permeability of the cell wall (Tukaj & Bohdanowicz 1995). The trilaminar structure of the cell wall contributes to its rigidity and mechanical resistance (Burczyk & Hesse 1981; Dunker and Wilhelm 2018). In the sheath, the presence of algaenans—aliphatic polymers containing long polymethylene chains with pyrrole rings substituted with amide and N-alkyl residues—is responsible for the particular endurance of cell walls in many strains of *Chlorophyta* (Domozych 2016, 2019; Domozych et al. 2012). Recently, it has been shown that the presence of algaenan substantially limits the release of soluble proteins into the extramembranous compartment in *Chlorella* and *Scenedesmus* microalgae (Zych et al. 2022). The other constituents which contribute to the integrity of the cell walls of *S. obliquus* are glucosamine-containing biopolymers and glycoproteins (Burczyk et al. 1999).

The growth of chlorococcalean algaenan-forming algae is accompanied by the accumulation of sporangial walls, the so-called cell walls of the mother cells (maternal cell walls (CWM)) in the medium. This feature is an important criterion in the taxonomy of *Chlorophyta* (Rashidi & Trindade 2018; Yamamoto et al. 2003). CWM is formed as a product of the eventual autolysis of the sporangial cell walls as a result of the division and size increase of the daughter cells (autospores) inside the maternal cells (sporangia) (Burczyk et al. 1970, 1971; Pickett-Heaps and Staehelin 1975). The accumulation of CWM in stationary phase cultures was associated with a decrease in accumulation of neutral sugars in cell walls, suggesting that the liberation of CWM is accompanied by partial deglycosylation of the cell wall components (Burczyk et al. 1995, 1999). Recently, CWM-associated linear polysaccharides of β-1,4-glucuronic acid and β-1,3-N-acetylglucosamine groups have been suggested to act as a virus-defence mechanism (Rashidi and Trindade 2018). Although the presence of certain enzymatic activities has been identified in CWM (Burczyk and Loos 1995), their protein composition has not been studied in detail. Similarly, the molecular mechanisms involved in the regulation of the formation and autolysis of sporangia, as well as the release of daughter cells, remain obscure.

Ubiquitin is a highly conserved, 76 amino acid, 8.5-kDa, globular molecule with a hydrophobic core composed of five fragments of a polypeptide chain linked by hydrogen bonds (Pickart and Eddins 2004). At the C-terminal, Gly76 site, ubiquitin attaches to the protein-activating enzyme (Kwon and Ciechanover 2017). Ubiquitin is present in the cells of all eukaryotes (Martín-Villanueva et al. 2021). In algae of the species *Chlamydomonas reinhardtii*, ubiquitin is present both in free form and in protein conjugates. It is mainly found in chloroplasts (51%), then in the cytosol (24%), nucleus (18%), and pyrenoids (7%). A significant amount has also been found near the cell membrane, and a small amount has been identified in the fraction containing mitochondria (Wettern et al. 1990).

Ubiquitin is involved in a number of processes fundamental to the cell, the most important of which is the intracellular degradation of proteins through the proteasomal pathway. It is also involved in DNA repair, the regulation of transcription and translation, cell cycle progression (Badarudeen et al. 2021), and the response of the organism to stress (Doroodian and Hua 2021; Xu and Xue 2019). In algae, ubiquitin is an important element involved in the stress response systems. In particular, heat treatment of *Chlamydomonas* cells increases the accumulation of ubiquitinated proteins with a molecular weight above 60 kDa, and the coordinated redistribution or decrease of other ubiquitinated proteins and free ubiquitin (Shimogawara & Muto 1989). Both heat shock and photoinhibition alter the ubiquitin conjugation pattern, indicating the role of ubiquitin in the response to heat and light stress in this algae (Wettern et al. 1990). More recently, it has been shown that protein ubiquitinylation is involved in the regulation of the acclimation of *Chlamydomonas* cells to elevated selenite concentrations (Vallentine et al. 2014) and plays a role in the nitric oxide-driven modulation of protein homeostasis in this organism (Kuo and Lee 2021).

The presence of ubiquitin has been demonstrated in the surface glycoproteins of eukaryotic cells (Sepulveda et al. 1996; Siegelman et al. 1986). More recently, ubiquitin and ubiquitin-processing enzymes have been shown to be essential, for such processes as signaling in the endosomal vesicle transport and cell-surface interactions (Clague and Urbé, 2017; Foot et al. 2017; Hicke 2001; Schnell and Hicke 2003). However, the role of ubiquitin in the extramembranous compartments of algal cells remains to be elucidated.

The *Scenedesmus obliquus* 633 strain, originally isolated and purified from the cooling circuit of the Zabrze power plant (Poland) (Jankowski 1964), has become a model organism for studying the structure and chemical composition of cell walls in *Chlorophyta.* In particular, CWM from this organism, accumulated in stationary phase of cultures, were extensively analyzed as a source of well-defined cell wall material (Burczyk 1973, 1979, 1987; Burczyk et al. 1970, 1971, 1981, 1999, 2014; Burczyk and Loos 1995; Voigt et al. 2014). It was found that *S. obliquus* 633 cell walls contain amino acids typical of proteins, but their content in CWM is different in comparison to that observed in the daughter cells (Burczyk et al. 1999). In addition, the maternal cell walls of *S. obliquus* 633 are rich in polyamines (Burczyk et al. 2014) and chaotrope-soluble glycoproteins with molecular masses > 150 kDa (Voigt et al. 2014). Furthermore, it has been shown previously that the multilayered cell wall of *Scenedesmus obliquus* 633 contains a polypeptide related to the cell-wall glycoprotein *gp3* of *Chlamydomonas reinhardtii* (Voigt et al. 2014). In this study, we show the presence of ubiquitin-related epitopes, ubiquitin-protein conjugates, and ubiquitin peptide signatures in a fraction of the maternal cell walls of *Scenedesmus obliquus* 633. Our results suggest the significance of ubiquitin-like proteins in the formation and processing of the cell walls during the lifecycle of this alga.

## Materials and methods

### Algal strain and culture conditions

Axenic cultures of the strain *Scenedesmus obliquus* 633, stored in the collection of the Department of Plant Physiology and Biochemistry of the Jagiellonian University, were grown in 2-L Erlenmeyer flasks containing 1 L of modified medium according to Kessler and Czygan (1970), supplemented with sucrose and glucose to final concentrations of 0.25% (w/v), as previously described (Burczyk et al. 1981). The medium (final pH ~ 6.3) was prepared as follows: 0.405 g KNO_3_, 0.235 g NaCl, 0.235 g NaH_2_PO_4_·2H_2_O, 0.18 g Na_2_HPO_4_·12H_2_O, 0.125 g, MgSO_4_·7H_2_O, 0.003 g FeSO_4_·7H_2_O, and 0.01 g CaCl_2_·7H_2_O were dissolved in 1 L of distilled water, and 1 mL of micronutrient solution, as described by Rippka et al. (1979), was added. Before inoculation, the media were sterilized at 121°C for 30 min. The cultures were maintained at 22°C and a humidity of 65%, under white fluorescent light illumination (Philips TL-D 18W/33–640), with a photon flux density of 60 µmol m^−2^ s^−1^, photoperiod: 16 h light:8 h darkness, and occasionally shaken.

### Isolation of maternal cell walls

At the stationary phase of cultures (approximately after 30 days), the cells and other insoluble materials were harvested by centrifugation for 20 min at 11,000* g* at 15°C.

The maternal cell walls were isolated according to the procedure as described in Burczyk et al. (1970, 1971). Briefly, the harvested sediment was suspended in 30 mL of distilled water in a 50-mL conical flask and centrifuged at 2500* g* for 10 min at 22°C. The supernatant was removed and the upper, pink-white layer of the sediment containing the maternal cell walls was collected with a spatula. As it contained intact cells, the remaining (green), bottom layer of the pellet, was discarded. The resulting material, enriched with maternal cell walls, was resuspended in the next volume of distilled water, vigorously vortexed, and centrifuged and the upper pellet layer was collected. This procedure was repeated several (usually five to six) times and the presence of intact cells in the collected material was checked each time by microscopic observations, until a cell-free specimen was obtained. The final specimens containing CWM from several independent *S. obliquus* 633 cultures were combined, dewatered via final centrifugation (13,000* g* for 5 min) and either kept frozen (− 30°C) or used immediately for further experimentation. All results presented in this study were based on CWM material isolated in three independent experiments (cycles of culture).

### Extraction of soluble proteins from CWM

In order to extract soluble protein fraction, purified CWM was resuspended in distilled water and incubated in a water bath for 60 min at 80°C with occasional shaking. The residual material was separated via centrifugation for 10 min at 13,400 g at room temperature. The collected supernatant was transferred to new tubes, freeze-dried under a vacuum at − 50°C, and stored deep-frozen until use. The protein concentrations in the extracts were recorded by the absorbance at 280 and 205 nm (Scopes 1974).

### Immunofluorescence staining

For immunofluorescence, aliquots of cell wall suspension in water were dispersed on microscope slides coated with chrome-alum gelatin (Pappas 1971) and kept overnight at 4°C. The staining was performed by indirect immunofluorescence according to the procedure described by Voigt et al. (2014) with some modifications.

Firstly, the slides were washed twice for 5 min in PBS to remove unbound material. Then, they were incubated for 60 min with a 1% (w/v) BSA (Sigma) solution in PBS to block nonspecific antibody binding sites, and subsequently washed three times with PBS for 5 min. The slides were then incubated overnight in a wet chamber at 4°C with the primary antibody (rabbit IgG anti-ubiquitin antibody, Sigma U-5379, 1:100 dilution in PBS or in some experiments Sigma SAB 136582, as indicated). In parallel, control specimens were incubated with 1:100 diluted rabbit plasma (zero specificity serum; IBSS Biomed, Kraków, Poland). After incubation, the slides were washed with PBS three times for 5 min. Next, the slides were incubated for 60 min with a secondary antibody (FITC-labeled goat anti-rabbit IgG, Sigma F0382, 1:48 dilution) and washed finally three times with PBS solution for 5 min. The specimens were mounted with a fluorescence quenching protection solution (10% (v/v) of PBS-buffered glycerol, supplemented with p-phenylenediamine at a final concentration of 0.1 mg/mL) under coverslips and examined under a NIB-620FL fluorescence microscope (Nexcope, Ningbo, China) in blue (maximum excitation wavelength of 450 nm) and visible light (bright field and phase contrast). The RGB DLT-Cam Viewer camera and the ToupTek/ToupView software (Nexcope) were used for the digital visualization. The immunofluorescence experiments were repeated three times with consistent results.

### SDS-PAGE and immunoblotting

The extract of soluble proteins from the maternal cell walls was denatured for 10 min at 80°C with a loading buffer (10% SDS, 500 mM DTT, 50% Glycerol, 500 mM Tris–HCL, 0.05% bromophenol blue). Then, the samples were centrifuged for 1 min, 13,000 g and the supernatant was collected. SDS-PAGE electrophoresis was performed using a polyacrylamide gel (Mini-Protean TGX Precast Gels, 4–15%, Bio-Rad Laboratories, No. 456–1083) for 60 min at a constant voltage 120 V. The gel underwent silver-staining according to Blum et al. (1987).

For immunoblotting, immediately after the electrophoresis, the gel was incubated in a carbonate buffer (3 mM Na_2_CO_3_, 10 mM NaHCO_3_) for 30 min. A PVDF membrane (0.45-μm pore size, Immobilon-P Transfer Membranes) was immersed in methanol for 15 s. The methanol was decanted, and the membrane was flooded with the carbonate buffer for 10 min. The proteins were electrotransferred from the gel to the membrane for 20 min at 15 V on a Trans-Blot Semi-Dry (Bio-Rad). After the electrotransfer, the membrane was washed with water and incubated for 30 min in blocking solution (0.2% Tween 20 in Tris-buffered saline (TBS)). Then, it was incubated with a primary rabbit anti-ubiquitin antibody diluted according to the producer’s recommendations in TTBS (0.05% Tween 20 in TBS) overnight at 4°C. Next, the membrane was washed three times for 5 min with TTBS solution. The secondary, goat, anti-rabbit, and horseradish peroxidase-conjugated antibodies (dilution 1: 10,000 in TTBS, Agrisera) were added and incubated for 1 h. The membrane was washed with TTBS (3 × 5 min) and incubated for 5 min in HRP substrate solution (SuperSignal West Pico Plus Chemiluminescent Substrate, Thermo Scientific). The membrane was placed in foil to remove excess moisture and visualized on a CCD BioSpectrum 800 imaging system densitometer (UVP, CA, USA) using the software VisionWorksLS, version 6.8.

For control experiments, the whole cell lysate from *Scenedesmus obliquus* 633 and *Chlamydomonas reinhardtii* (SAG 11-32b) were prepared as follows: 20 ml of algal culture suspension was harvested by centrifugation for 20 min at 11,000 g at 15°C. The pellet was resuspended directly in 2 mL of the loading buffer and disrupted for 5 min by intensive shaking with glass beads (100 µm). The fraction of soluble proteins was obtained by a centrifugation of whole cell lysate for 30 min, 16,000 g, at 4°C. Both the supernatant containing soluble proteins and the whole cell lysate were denatured for 10 min at 80°C and subjected to SDS-PAGE and immunoblotting as described above. The SDS-PAGE and immunoblotting experiments were repeated three times using protein extracts from independent CWM isolates.

### Protein tryptic digestion and LC–MS/MS analysis

For LC–MS/MS analysis, a combined CWM material isolated from several independent *S. obliquus* 633 cultures was extracted as described above. The concentrated water extract of soluble proteins from the maternal cell walls was analyzed using LC–MS/MS as described previously (Pinski et al. 2021). In brief, the sample was suspended in a urea solution (8 M urea in 50 mM ammonium bicarbonate) and prepared according to the FASP protocol (Wiśniewski et al. 2009). The proteins were reduced with dithiothreitol (DTT) (50 mM, 15 min, RT), transferred to a spin column (30-kDa membrane cut-off, VIVACON 500, Sartorius Stedim Biotech GmbH, Göttingen, Germany), and alkylated in the dark with iodoacetamide (54 mM, 20 min, RT). After washing, the proteins were digested with trypsin (Promega) at an enzyme-to-protein ratio of 1:60 overnight at 37 °C. The resulting peptides were washed out with 50 mM ammonium bicarbonate and 0.5 M NaCl, collected and vacuum dried. The sample was analyzed using nanoLC-MS/MS on a Q-Exactive mass spectrometer coupled to an UltiMate 3000 RSLCnano System (Thermo Fisher Scientific) in duplicate. The peptide mixture was suspended in a loading buffer (2% acetonitrile with 0.05% trifluoroacetic acid), loaded onto a trap column (Acclaim™ PepMap™ 100 C18 HPLC Columns, ID 75 µm, 20 mm length, particle size 3 μm, pore size 100 Å) in 0.05% TFA and 2% acetonitrile at a flow rate of 5 µL/min and resolved on an C18 analytical column (Acclaim™ PepMap™ RLSC C18, ID 75 μm, length 500 mm, particle size 2 μm, pore size 100 Å) at a flow rate of 250 nL/min. The peptides were eluted with a 240-min gradient from 2 to 40% of acetonitrile with 0.05% formic acid. The mass spectrometric measurements were performed in the data-dependent mode using the Top12 method. The MS and MS/MS spectra were acquired at resolutions of 70,000 and 17,500, respectively.

The RAW files from the two replicates were processed together using the platform Proteome Discoverer (v.1.4, Thermo Fisher Scientific) and searched against the NCBI database (https://www.ncbi.nlm.nih.gov/) three times: with *Scenedesmaceae* taxonomy restriction (release June 2021, 25,074 sequences), with *Chlorophyceae* taxonomy restriction (release July 2021, 390,876 sequences), and with *Chlamydomonas reinhardtii* taxonomy restriction (release July 2021, 31,451 sequences) using locally the search engine MASCOT (v.2.5.1, Matrix Science). The following parameters were applied: fixed modification—cysteine carbamidomethylation; variable modifications—methionine oxidation and protein N-terminal acetylation; the peptide mass tolerance—10 ppm; fragment mass tolerance—20 mmu. Only tryptic peptides with up to one missed cleavage were considered. The false discovery rate for peptide-spectrum matches was set to 0.01 using Percolator.

### Computational analysis of proteins

The subcellular locations of the proteins were identified and the signal peptides were predicted using TargetP 2.0 (https://services.healthtech.dtu.dk/service.php?TargetP-2.0), as described by Armenteros et al. (2019). The signatures of the conserved protein domains were assessed using the software ScanProsite (de Castro et al. 2006). The transmembrane helices were identified using the tool TMHMM (https://services.healthtech.dtu.dk/service.php?TMHMM-2.0 (Krogh et al. 2001).

## Results and discussion

### Immunochemical detection of ubiquitin-like epitopes in CWM

**Fig. 1:**
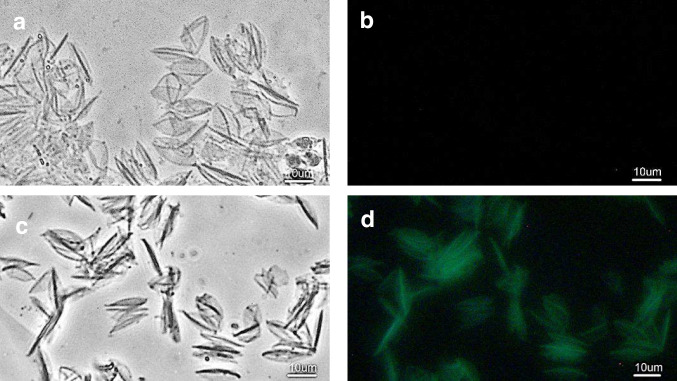
Immunofluorescence analysis of the purified maternal cell walls from *Scenedesmus obliquus 633* strain. A control specimen **a**, **b** incubated with rabbit plasma as a primary antibody and a test specimen **c**, **d** incubated with a primary anti-ubiquitin antibody/FITC-labeled secondary antibody. Specimens viewed in **a**, **c** phase contrast, **b**, **d** blue light. Scale bars: 10 μm

As observed in light microscopy, the isolated CWM of *Scenedesmus obliquus* 633 has a characteristic, fusiform shape, which is especially distinguishable when using phase contrast optics (Fig. 1a). Fluorescence attributable to the binding of polyclonal anti-ubiquitin antibodies was observed in CWM isolated from stationary phase cultures of the strain *S. obliquus* 633. The fluorescence image reflected the image of CWM precisely, as observed in phase contrast (Fig. 1c, d). The control specimens, in which the normal rabbit serum was used as a primary antibody, showed no fluorescence attributable to the binding of the secondary antibody (Fig. 1a, b). Similarly, a specific binding of polyclonal antibody directed to the C-terminal part of the ubiquitin was observed in isolated CWM (see Supplementary Fig. S1). These results indicate that the ubiquitin-like epitopes are present on the surface of CWM of *S. obliquus* 633.

As previously reported, polyclonal antibodies specific to both deglycosylation products of the *Chlamydomonas reinhardtii* cell wall polypeptides and glycosylated GP3B cell wall-associated protein reacted specifically with the CWM of *Scenedesmus obliquus* 633 (Voigt et al. 2014). In *Candida albicans*, the polyclonal anti-ubiquitin serum was used to identify ubiquitin-like epitopes of putative receptor proteins accompanied by the cell walls of this fungus (Sepulveda et al. 1996). As both sequence- and structure-based epitopes might be recognized by polyclonal antisera (Lipman et al. 2005), our results suggest that (poly)peptides containing conserved aminoacid sequences similar to ubiquitin and/or ubiquitin-related structures are co-localized with the surfaces of CWM of *S. obliquus* 633.

**Fig. 2:**
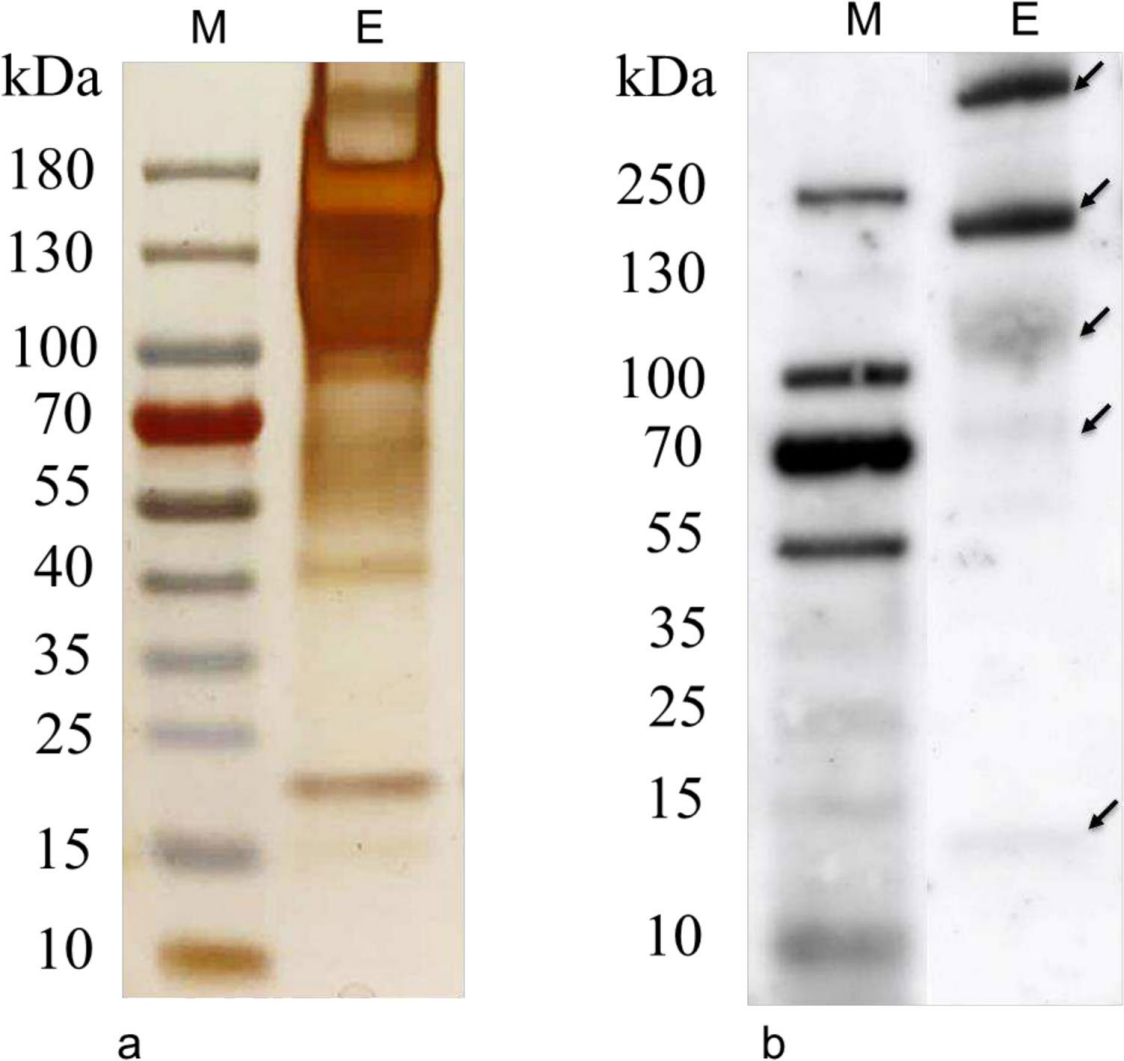
SDS-PAGE **a** and immunoblotting **b** analysis of the protein extract of maternal cell walls incubated with anti-ubiquitin antibody. Ubiquitin conjugates as detected in immunoblotting are marked with arrows

To describe further the nature of epitopes recognized by anti-ubiquitin rabbit serum, soluble proteins were extracted from CWM with water at 80°C. Under these conditions, an efficient elution of proteins from the cell wall material as isolated from several algal species, including those forming trilaminar cell walls, has been observed (Zych 2001). In fact, a number of polypetides with an apparent molecular mass of c. 15 kDa to > 180 kDa appeared on the gradient SDS-PAGE gel after silver-staining (Fig. 2a). Although these proteins represented a broad range of apparent molecular weights, the accumulation of polypeptides was different in comparison to this, which was observed both in the whole cell lysate and in the fraction of soluble proteins from *S. obliquus* 633 cells (compare Supplementary Fig. S2a). In particular, an abundant band with an apparent molecular mass of c. 180 kDa was detected (Fig. 2a).

Extraction with high concentrations of chaotropic salts has previously been applied to solubilize hydroxyproline-rich glycoproteins, associated with the cell walls of *Chlamydomonas reinhardtii* (Voigt 1985, 1988; Voigt et al. 2009). In particular, it has been shown that the chaotrope-soluble cell wall glycoprotein GP1, with a molecular mass of c. 272.4 kDa, was the only hydroxyproline-enriched polypeptide that occurs in vegetative *C. reinhardtii* cells (Voigt et al. 2009). The LiCl extracts from the mother-cell walls isolated from *S. obliquus* 633 contained exclusively several high-molecular-weight components with apparent molecular masses > 150 kDa (Voigt et al. 2014). In contrast, the water extract from this material at 80°C, as obtained in this study, was a composition of a number of polypeptides representing a broad range of apparent molecular masses. This result indicates that the elevated temperature may increase the solubility of CWM-associated proteins in water, e.g., as a consequence of the increase in the negative surface charges (Kramer et al. 2012), and/or it might reduce the adsorption of polypeptides to the polysaccharide/polycarbonate matrix in the cell wall by a disruption of hydrogen bonds and other molecular interactions (Mcqueen-Mason and Cosgrove 1994; Wohlert et al. 2022). Thus, the abundant, high molecular mass polypeptides, as observed in our water extracts, may reflect, at least partially, the CWM components previously identified via chaotrope solubilization (Voigt et al. 2014).

Immunoanalysis of water-soluble proteins extracted from CWM and resolved by SDS-PAGE showed the presence of only a few peptides reactive with polyclonal anti-ubiquitin serum (Fig. 2b). In contrast, both in the whole cell extract and in the soluble protein fraction from *Scenedesmus obliquus* 633 cells, the anti-ubiquitin serum was able to recognize many proteins with molecular masses c. 15 kDa to > 180 kDa (see Supplementary Fig. S2b). Additionally, in whole-cell extracts from *Chlamydomonas reinhardtii*, these antibodies bound mainly to polypeptides of lower molecular weight, including proteins of apparent molecular weights of 18–31 kDa, whose ubiquitination has previously been shown in this algae (Shimogawara and Muto 1989) (see Supplementary Fig. S3). These results indicate that the antibody reactivity is specific, and polypeptides recognized in CWM extract may contain, in fact, ubiquitin-like epitopes.

The most prominent antibody-binding polypeptides as identified in the CWM extract had apparent molecular masses of c. 200 and > 250 kDa. The less marked polypeptides were observed at c. 12, 70, and 120 kDa. Interestingly, polypeptides of the most abundance in the CWM extract (> 100 kDa) reacted specifically to anti-ubiquitin antibodies. This result shows that ubiquitin-like epitopes as recognized by anti-ubiquitin polyclonal antibodies are connected with polypeptides of molecular mass far exceeding the molecular mass of the monomeric form of ubiquitin (8.5 kDa). Consequently, these polypeptides may represent the (poly)ubiquitynylated proteins of higher molecular mass and/or proteins containing ubiquitin-related epitopes as recognized by the polyclonal anti-ubiquitin serum, in their primary structure (Sepulveda et al. 1996). These conjugates might be products of the post-translational modifications of existing proteins by not-yet-defined ubiquitin ligases (Zheng and Shabek 2017) and/or proteins containing domains structurally related to ubiquitin (Collins and Goldberg 2020; Herrmann et al. 2007). Additionally, the presence of chimeric genes, including ubiquitin-like coding sequences fused to those encoding proteins of different properties, has recently been demonstrated in algae of the divisions *Cryptophyte* and *Chlorarachniophyte* (Sibbald et al. 2019). Although such genetic fusions have not yet been identified in *Chlorophyta*, the presence of similar structures among CWM-associated proteins cannot be ruled out.

### Identification of CWM proteins using LC–MS/MS

To identify the CWM-associated proteins, the concentrated water extract from CWM underwent tryptic digestion as described in the “Materials and methods” section. The resulting polypeptides were then separated using LC accompanied by MS/MS spectrometry. These MS/MS spectra were assigned to polypeptide sequences using NCBI databases, containing protein sequences as deduced from known whole algal genomes, with restrictions to the taxonomies *Scenedesmaceae*, *Chlorophyceae*, and *Chlamydomonas reinhardtii*. The statistics of the protein identification are shown in Table 1. Of over 150,000 independent mass spectra, only c. 0.1% were successfully assigned to peptides. Moreover, the number of identified peptides was remarkably lower in comparison to the number of spectra assigned to peptides annotated in each database analyzed. These results indicate that most of the protein/peptide material as extracted from CWM contains chemical modifications that prevent its direct identification using mass spectrometry. It has previously been shown that insoluble hydroxyproline-containing glycoproteins are constituents of the cell walls of higher plants (Cassab 1998) and green algae (Voigt et al. 1994), including *Chlamydomonas reinhardtii* (Imam et al. 1985; Roberts et al. 1985). Thus, this result suggests that extraction with water at an elevated temperature may, at least partially, promote the solubilization of post-translationally modified CWM protein components in *Scendesmus obliquus* 633.

**Table 1:** Numbers of collected spectra, spectra assigned to peptides, identified peptides, and identified proteins for each database

NCBI database	Number of collected spectra	PSM (peptide spectrum matching)—spectra assigned to peptides	Number of identified peptides	Number of proteins identified in the database	% of assignment
*Scenedesmaceae*	156706	129	80	58	0.08
*Chlorophycaeae*	156706	61	43	37	0.04
*Chlamydomonas reinhardti*	156706	37	24	18	0.02

**Fig. 3:**
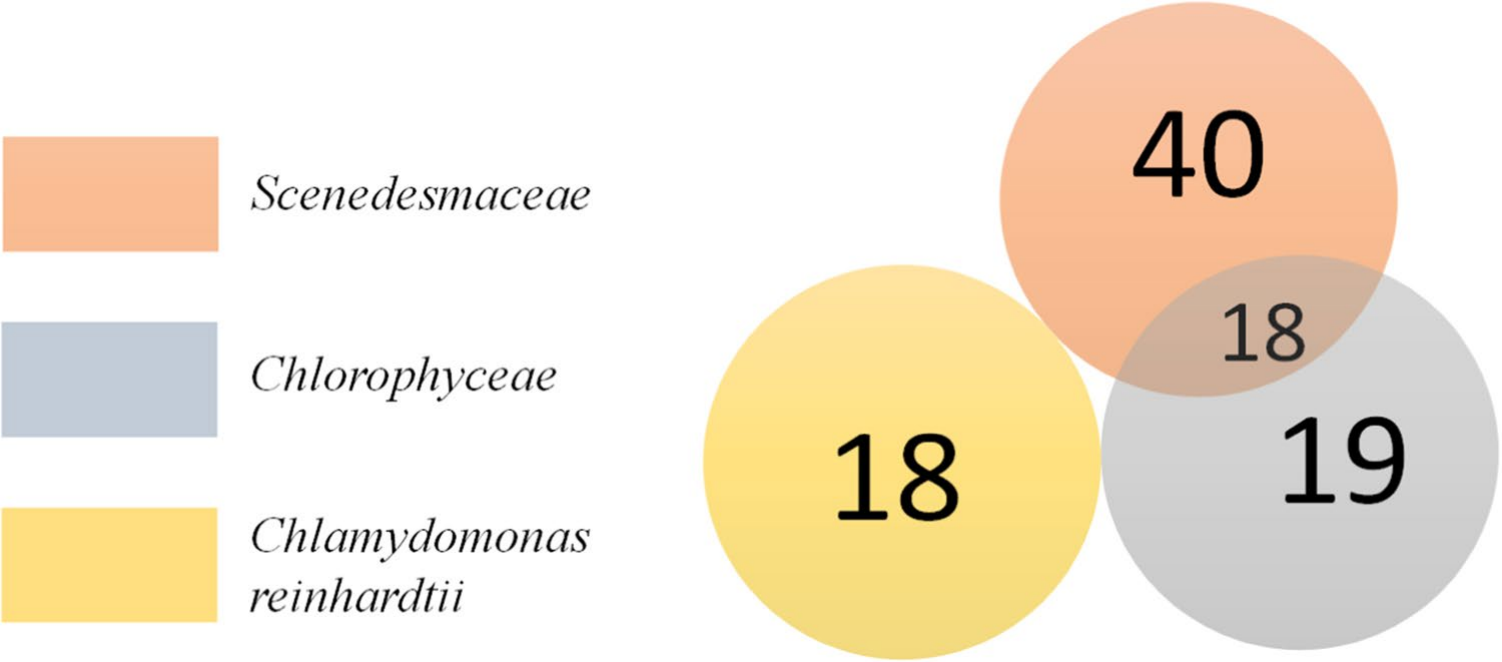
Venn diagram showing the number of proteins identified in *Scenedesmus obliquus* 633 strain depending on the database used for identification. See text for details

A search of the databases found a total of 113 records corresponding to 95 identified protein signatures, including 58 records in the *Scenedesmaceae* database, 37 records in the *Chlorophyceae* database, and 18 records in the database of *Chlamydomonas reinhardtii*. A Venn diagram, presenting the relationship between positive matches, is shown in Fig. 3. As can be seen, 18 identified proteins occurred in both the *Scenedesmaceae* and *Chlorophyceae* databases, whereas proteins identified in the *Chlamydomonas* database formed a set separate from the other two. The full list of records and identified proteins is given in a Supplementary Table S1.

Of the identified proteins, 30 are hypothetical ones, not assigned so far to any cellular structure/function. An analysis using the tool Target P demonstrated the presence of N-terminal signal peptide for the putative ER/extracellular transport in 13 proteins, chloroplast/thylakoid transfer signatures in 12 proteins, and mitochondrial transfer peptide in 6 proteins. All remaining proteins expressed none of the compartment-specific signatures and were deemed to be localized in the cytoplasm.

Of the proteins identified on the basis of peptide signatures in the trypsin-treated extracts that are listed in Supplementary Table S1, three positive matches pointed to the ubiquitin-related proteins. These include KAG2432108.1, a hypothetical protein from *Chlamydomonas incerta*, KAF8061345.1, UBQ10 from *Scenedesmus* sp., and GIM07828.1 hypothetical protein from *Volvox reticuliferus*. A ScanProsite analysis revealed the presence of conserved ubiquitin domains in all these proteins. The amino acid sequences of the peptide signatures related to these proteins are shown in Table 2. The representative chromatogram and a deduced aminoacid sequence of UBQ10 are shown in Supplementary Fig. S4. Interestingly, the sequences of all these peptides show a high level of similarity to those identified previously in the analysis of plant proteomes (K. Zhang et al. 2006; Zhou et al. 2016). This result indicates that the structure of these proteins in *S. obliquus* can be conserved relative to their plant orthologs.

**Table 2:** Identified ubiquitin-like proteins and peptide signature sequences

Accession	Description	Peptide signature sequences
KAF8061345.1	UBQ10 [*Scenedesmus* sp. PABB004]	TITLEVESSDTIENVK
		ESTLHLVLR
		TLADYNIQK
GIM07828.1	hypothetical protein [*Volvox reticuliferus*]	TITLEVESSDTIENVK
		ESTLHLVLR
KAG2432108.1	hypothetical protein [*Chlamydomonas incerta*]	TITLEVESSDTIENVK
		ESTLHLVLR
		TLADYNIQK

The open character of the cell wall compartment is a serious hindrance to the isolation of cell wall fractions free of intracellular contaminants (Albenne et al. 2013, 2014; Regente et al. 2017). However, the limited contribution of lipophilic proteins as identified by LC–MS/MS proteomic analysis supports the conclusion that most of the residual cell-membrane and other cellular components were washed out prior to protein extraction. As can be seen from Supplementary Table S1, the presence of peptide signatures specific to proteins with plastid, mitochondrial, and cytoplasmic localizations suggests that a water-extracted pool of CWM polypeptides may contain, at least to some extent, the protein material that originated from the protoplast compartments of sporangium. Therefore, an observation of the ubiquitin signatures in the proteomic analysis of the water-extracted cell wall protein pool cannot unequivocally confirm the presence of ubiquitin as a *bona fide* component of cell wall proteome in *S. obliquus* 633. However, the binding of polyclonal anti-ubiquitin antibodies to CWM-associated high-molecular mass polypeptides, accompanied by a subsequent identification of relevant signatures, supports the conclusion that ubiquitin-like domains are, in fact, present in the extramembranous compartment of this algae.

To assess whether the water-extracted fraction from CWM may contain proteins that are specifically oriented towards the extramembranous compartment, we analyzed the proteins identified according to criteria recently applied in identifying *bona fide* plant cell wall proteome components. These include the presence of a N-terminal signal peptide, no ER signal sequence, and no more than two transmembrane domains detected (Clemente et al. 2022). On the basis of computational predictions, we found that 12 proteins jointly matched these criteria. The full list of proteins identified is given in Table 3.

**Table 3:** Proteins selected on the basis of bioinformatic analysis as *bona fide* components of the wall proteome. Database: (A) *Scenedesmaceae* (accessed in June 2021, 25074 sequences, https://www.ncbi.nlm.nih.gov/), (B) *Chlorophyceae* (accessed in July 2021, 390876 sequences)

Accession	Database	TargetP	Prosite	TMHMM—2.0	Description	Score	Coverage	# Proteins	# Unique Peptides	# Peptides	# PSMs	# AAs	MW [kDa]
KAF6251784.1	A,B	S	APPLE, PS00495; apple domain	# KAF6251784.1 number of predicted TMHs: 0	Hypothetical protein [*Scenedesmus* sp. NREL 46B-D3]	116,68	0,7	3	1	1	2	1433	148,6
KAF6256986.1	A,B	S	PS00698 GH9_3 glycosyl hydrolases family 9 (GH9) active site signature 3	KAF6256986.1 number of predicted TMHs: 2	Six-hairpin glycosidase-like protein [*Scenedesmus* sp. NREL 46B-D3]	70,75	1,42	1	1	1	1	988	105,2
KAF6261957.1	A,B	S	PS50026 EGF_3 EGF-like domain profile:	KAF6261957.1 number of predicted TMHs: 0	Hypothetical protein [*Scenedesmus* sp. NREL 46B-D3]	87,37	0,99	3	2	2	3	2214	229,4
KAF6264072.1	A,B	S	PS01248 EGF_LAM_1 laminin-type EGF-like (LE) domain signature:	KAF6264072.1 number of predicted TMHs: 0	Hypothetical protein [*Scenedesmus* sp. NREL 46B-D3]	109,74	1,89	1	1	1	2	741	76,3
KAF6264724.1	A,B	S	No hit!	# KAF6264724.1 number of predicted TMHs: 0	Hypothetical protein [*Scenedesmus* sp. NREL 46B-D3]	59,84	3,57	1	1	1	2	252	28,4
KAF6266133.1	A,B	S	PS50842 EXPANSIN_EG45 Expansin, family-45 endoglucanase-like domain profile	KAF6266133.1 number of predicted TMHs: 0	RlpA-like double-psi beta-barrel-protein domain-containing protein-containing protein [*Scenedesmus* sp. NREL 46B-D3]	52,41	5,91	1	1	1	1	220	24,7
KAF8059426.1	A	S	PS00099 THIOLASE_3 thiolases active site:	KAF8059426.1 number of predicted TMHs: 0	BGLU42 [*Scenedesmus* sp. PABB004]	22,96	0,76	1	1	1	1	1046	109,9
KAF8060350.1	A	S	No hit!	KAF8060350.1 number of predicted TMHs: 0	Hypothetical protein [*Scenedesmus* sp. PABB004]	37,99	0,98	1	1	1	2	817	80,3
KAF8064679.1	A	S	PS50191 CRAL_TRIO CRAL-TRIO lipid binding domain profile:	KAF8064679.1 number of predicted TMHs: 0	pitC [*Scenedesmus* sp. PABB004]	29,63	0,81	1	1	1	1	1486	158,9
KAF8072813.1	A	S	PS50842 EXPANSIN_EG45 Expansin, family-45 endoglucanase-like domain profile:	KAF8072813.1 number of predicted TMHs: 0	EXPA15 [*Scenedesmus* sp. PABB004]	116,19	1,89	1	1	1	2	477	52,4
KAF8073058.1	A,B	S	PS51689 SAM_RNA_A_N6_MT rRNA adenine N(6)-methyltransferase family profile: PS00698 GH9_3 glycosyl hydrolases family 9 (GH9) active site signature 3:PS01131 RRNA_A_DIMETH ribosomal RNA adenine dimethylases signature:	KAF8073058.1 number of predicted TMHs: 0	celI [*Scenedesmus* sp. PABB004]	96,66	0,86	2	1	1	2	1621	167,8
XP_013899571.1	B	S	PS50842 EXPANSIN_EG45 Expansin, family-45 endoglucanase-like domain profile:	# XP_013899571.1 number of predicted TMHs: 0	Hypothetical protein [*Monoraphidium neglectum*]	101,69	4,17	2	1	1	2	216	24,4

According to the ScanProsite predictions, three proteins have an expansin domain signature, two proteins bear glycosyl hydrolase active sites, three are putative lipid binding proteins, and two proteins express EGF-like domains. Two proteins contained no specific domains. Interestingly, all of these proteins are matched with the *Scenedesmaceae* and/or *Chlorophyta* databases.

These results are in accordance with previous findings, both experimental and computer-based, that deal with the protein composition of algal and plant cell walls. In particular, several glycosidase activities have been experimentally identified in CWM of *S. obliquus* 633 (Burczyk and Loos 1995). Additionally, a glycosyltransferase involved in arabinofuranose transfer and extensins specific to the cell wall has been found in *Arabidopsis thaliana* and predicted in *Characean* algae (Møller et al. 2017). Extensins are a broad family of hydroxyproline-rich glycoproteins unique to the plant kingdom (Domozych 2016; Lamport et al. 2011; Showalter et al. 2010). Recent bioinformatic analysis has demonstrated that green algae may contain a set of chimeric extensin-like proteins (Liu et al. 2016). Thus, the identification of laminin and EGF-like domains among putative members of CWM proteome may suggest the presence of similar glycoproteins of unknown character in CWM of *S. obliquus* 633. Similarly, proteins bearing the PAN/Apple domains have previously been found to interact with carbohydrates (Tordai et al. 1999).

Expansins are plant proteins that demonstrate cell wall loosening activity. Their N-terminal domain is homologous to the catalytic domain of proteins in the glycoside hydrolase family 45 (Sampedro and Cosgrove 2005). The presence of three putative low-molecular-weight expansins suggests that these proteins might be involved in the enlargement and/or breakdown of CWM during the maturation of sporangia in the lifecycle of *S. obliquuus* 633.

In eukaryotic cells, extracellularly secreted ubiquitin has been found to play a role in the regulation of various processes, including cell growth and survival (Jackson et al. 2018; Zhang et al. 2019), regulation of immune response (Sujashvili 2016), and modification of surface receptors (Daino et al. 2000). Interestingly, a trans-membrane ubiquitin ligase E3 that co-expressed with secondary wall-associated genes has recently been shown to localize in plant plasma membranes. This ligase activity has been proposed to be involved in secondary wall formation and programmed cell death in *Arabidopsis* inflorescence stems (Noda et al. 2013). Similarly, in the mushroom *Volvariella volvacea*, the process of cryogenic autolysis has been correlated with the specific expression of the ubiquitin-conjugating enzyme E2 (UBE2) (Gong et al. 2016). Thus, the results presented in this study may suggest the involvement of ubiquitin-dependent signaling in such processes as formation and/or autolysis of sporangial cell walls. Although many identified proteins contain the signal peptide, which suggests their secretion to the extramembranous compartment via conventional ER-Golgi transport, other secretion pathways, as recently identified in plant cells, such as Golgi-bypass, multivesicular bodies-plasma membrane fusion, vacuole–plasma membrane fusion, or EXPO-mediated secretion pathway, cannot be excluded (see: Chung and Zeng 2017, for a review). In fact, both ubiquitin and subunits of proteasome have also been identified in plant extracellular vesicles (Regente et al. 2017). Early observations using transmission electron microscopy revealed various membraneous vesicles with different dimensions and densities in the space between the cell wall and the plasmalemma in several *Chlorella* mutants (Burczyk and Hesse 1981). The recent confirmation of the formation of extracellular vesicles in plants and microalgae (Boevink 2017; Picciotto et al. 2022; Regente et al. 2017; Rutter and Innes 2017) supports the explanation that the ubiquitin-like proteins as detected in this study in CWM from *S. obliquus* 633, might be, at least partially, products of extracellular transport via a microvesicle-driven mechanism.

## Conclusion

In summary, immunofluorescence microscopy, immunoblotting analysis, and LC–MS/MS proteomics collectively demonstrate the presence of ubiquitin-like epitopes, ubiquitin-specific peptide signatures, and several ubiquitin conjugates of higher molecular mass associated with the maternal cell walls of *Scenedesmus obliquus* 633. The presence of CWM-associated ubiquitin-like proteins suggests that the protein components of the extramembranous compartment may share considerable structural similarity to surface proteins previously found in other eukaryotes. More specifically, the detection of ubiquitin-like proteins and ubiquitin conjugates in sporangial cell walls points to the significance of the protein ubiquitination system in the regulation of the formation and/or autolysis of sporangia in *Scenedesmaceae*. Further research is needed, including the detailed analysis of proteins from the cell wall fraction of daughter cells, to elucidate the origin and function of ubiquitin-like proteins in the extramembranous compartment of *Scenedesmus* and other *Chlorophyta*.

## Supplementary information

Below is the link to the electronic supplementary material.

Supplementary file1 (DOCX 2879 KB)

## Data Availability

Data supporting the findings of this study are available from the corresponding author [PM] on request.
