# Supplementary material for: Ubiquitin-like and ubiquitinylated proteins associated with the maternal cell walls of Scenedesmus obliquus 633 as identified by immunochemistry and LC–MS/MS proteomics
Source: Protoplasma. 2024 Oct 4;262(2):299–312. doi: 10.1007/s00709-024-01994-3 (PMC11839794; doi:10.1007/s00709-024-01994-3)
Supplement: Supplementary file 1 — Supplementary file1 (DOCX 2879 KB) [file 709_2024_1994_MOESM1_ESM.docx]

ELECTRONIC SUPPLEMENTARY MATERIAL

Ubiquitin-like and ubiquitinylated proteins associated with maternal cell walls of *Scenedesmus obliquus* 633 as identified by immunochemistry and LC-MS/MS proteomics

Justyna Kowalczyk*^1,2^*, Kinga Kłodawska*^1^,* Maria Zych*^3^*, Jan Burczyk***^3,4^***, Przemysław Malec*^1,*^*

*^1^Department of Plant Physiology and Biochemistry, Faculty of Biochemistry, Biophysics and Biotechnology, Jagiellonian University, 30-387, Kraków, Poland*

*^2^Doctoral School of Exact and Natural Sciences, Jagiellonian University, 30-348 Kraków, Poland*

***^3^****Department of Pharmacognosy and Phytochemistry, Faculty of Pharmaceutical Sciences in Sosnowiec, Medical University of Silesia, Katowice, Jagiellońska 4, 41-200 Sosnowiec, Poland*

*^4^Laboratory of Biotechnology, Puńcowska 74, 43-400 Cieszyn, Poland*

**

**Supplementary Fig. S1** Immunofluorescence analysis of purified maternal cell walls from *Scenedesmus obliquus* 633 strain. A control specimen (a, b) incubated with rabbit plasma as a primary antibody viewed with 1000x magnification. A test specimen (c, d) incubated with a primary anti-ubiquitin antibody (Sigma SAB 136582)/FITC-labeled secondary antibody viewed with 1000x magnification. Specimens viewed in: a, c – bright field, b, d- blue light. Scale bars: 10 μm.

*
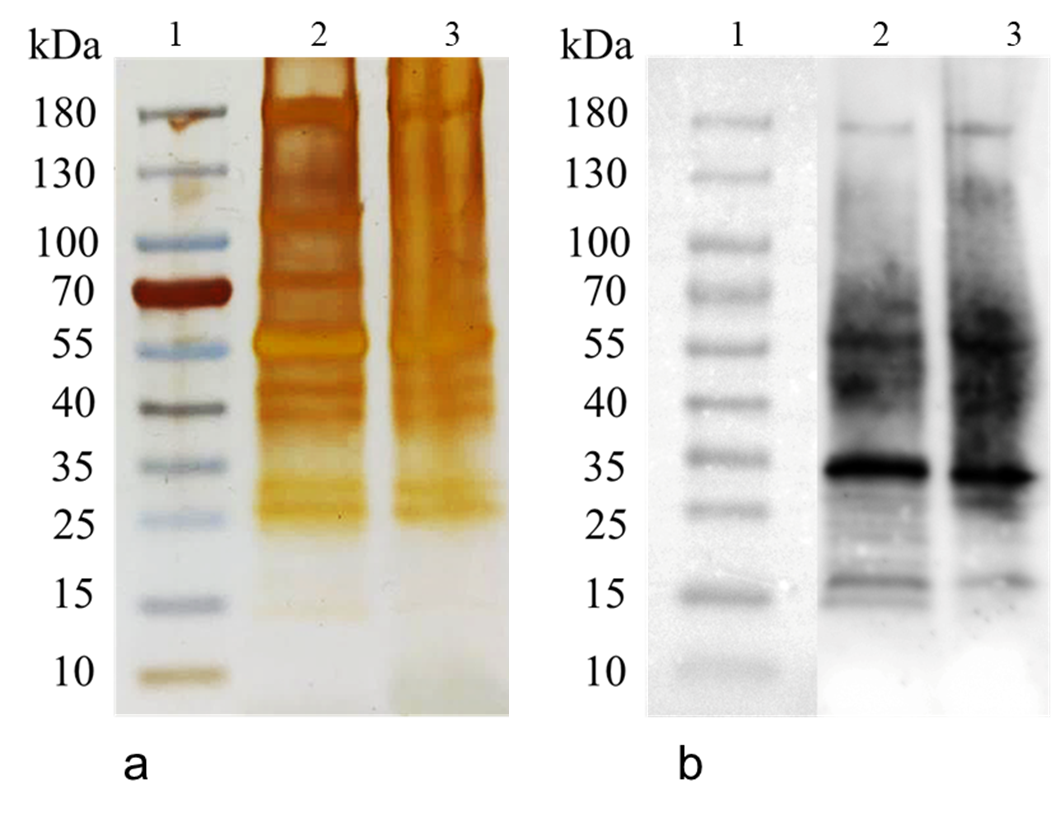
*

**Supplementary Fig. S2.** SDS-PAGE (a) and immunoblotting (b) analysis of the soluble fraction and whole cell lysates of *Scenedesmus obliquus* 633 incubated with anti-ubiquitin antibody. Lane 1- marker; Lane 2 - soluble fraction; Lane 3- whole cell lysates*.* See text for details.

*
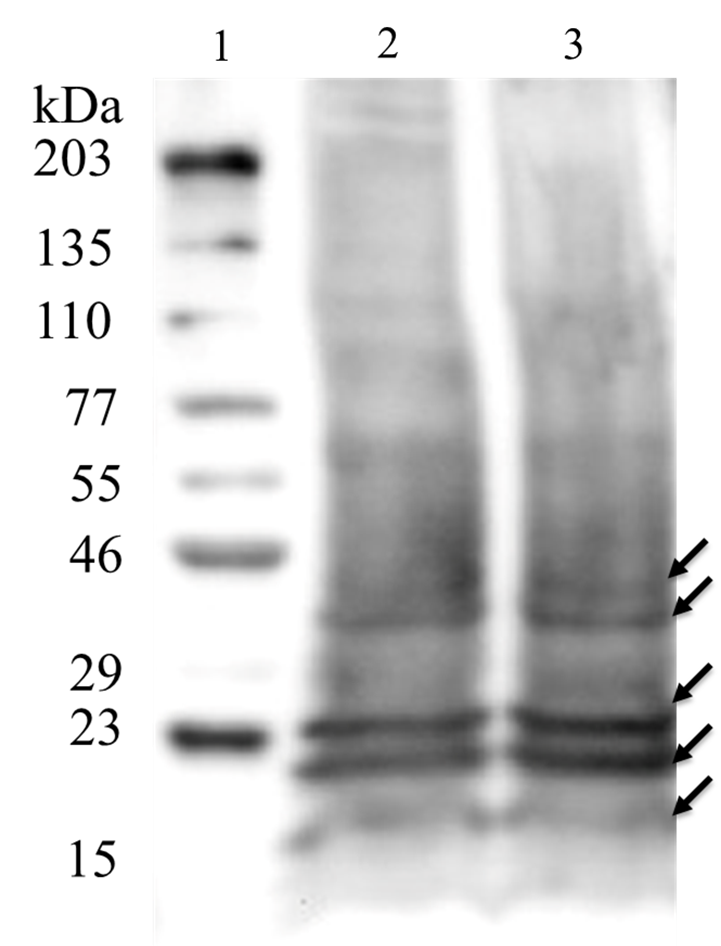
*

**Supplementary Fig. S3.** Immunoblotting analysis of the whole cell lysate from *Chlamydomonas reinhardtii*, (strain 11–32b, SAG collection, Goettingen, Germany), with anti-ubiquitin antibodies used in this work. Main peptides containing ubiquitin-like epitopes are marked with arrows. Lane 1- marker, Lane 2- *Chlamydomonas reinhardtii* grown under standard conditions (Sager-Granick medium, 35–40 μmol photons m^–2^ s ^–1^ on a shaker at 21°C ), Lane 3 - *Chlamydomonas reinhardtii* grown under standard conditions and subsequently incubated for 30 min at 40^o^C. Results are in accordance with previously published data (Shimogawara 1989).

**A**


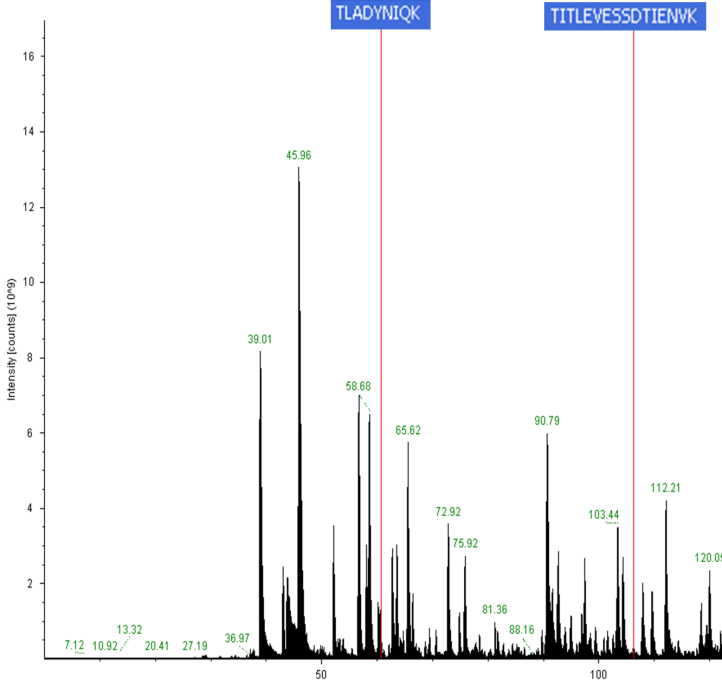


**B**


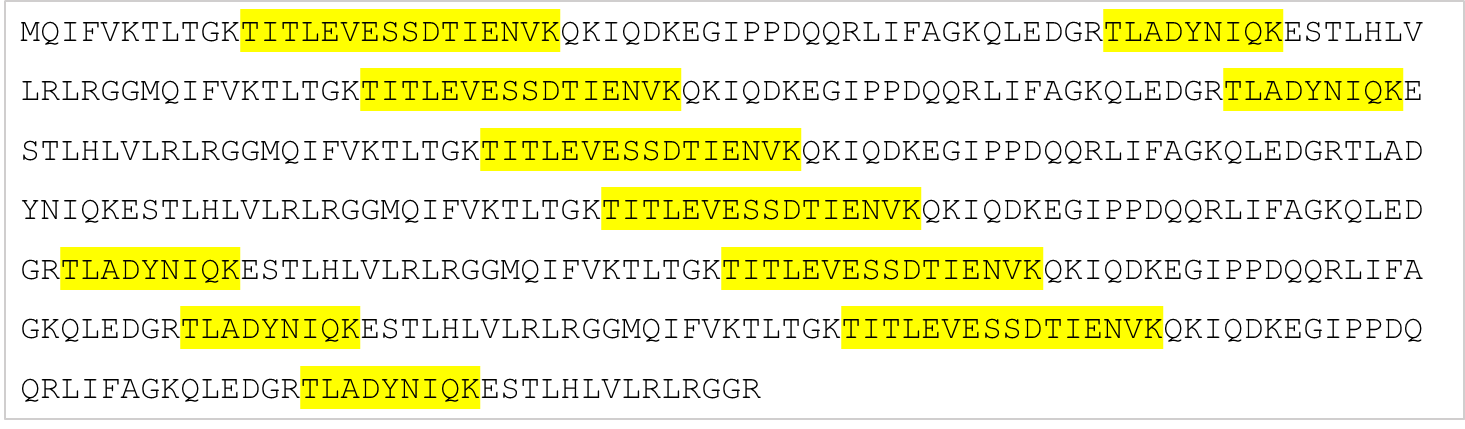


**Supplementary Fig. S4.** Base Peak LC Chromatogram (A) and a sequence of UBQ10 protein (B). Red lines show the positions of peptide signatures of UBQ10 protein. Ubiquitin-like domains marked in yellow.

**Supplementary Table S1.** Identified proteins, with database accession numbers, protein names, mass spectrometric analysis parameters, sequences based on which they were identified and theoretical molecular weights. Database: A- *Scenedesmaceae* (accessed on June 2021, 25074 sequences, <https://www.ncbi.nlm.nih.gov/>), B- *Chlorophyceae* (accessed on July 2021, 390876 sequences, <https://www.ncbi.nlm.nih.gov/>), C- *Chlamydomonas reinhardtii* (accessed on July 2021, 31451 sequences, <https://www.ncbi.nlm.nih.gov/>). TargetP: 0- no targeting peptide; S- N-terminal signal peptide; M- mitochondrial transit peptide; C-chloroplast transit peptide; T- thylakoidal lumen composite transit peptide.

| **Target P** | **Database** | **Accession** | **Description** | **Score** | **Coverage** | **# Proteins** | **# Unique Peptides** | **# Peptides** | **Sequence** | **# PSMs** | **MW [kDa]** |
| --- | --- | --- | --- | --- | --- | --- | --- | --- | --- | --- | --- |
| C | A,B | KAF6263730.1 | 33kDa oxygen evolving protein of photosystem II [Scenedesmus sp. NREL 46B-D3] | 301,38 | 19,73 | 2 | 1 | 4 | GTLDAFGGEFVVPSYR | 10 | 30,7 |
|  |  |  |  |  |  |  |  |  | VDAATGEIAGVFESIQPSDTDLGAK |  |  |
|  |  |  |  |  |  |  |  |  | VPFLFTVK |  |  |
|  |  |  |  |  |  |  |  |  | GSAVFSVAK |  |  |
| 0 | A | XP_013890557.1 | Histone H4 [Monoraphidium neglectum] | 256,50 | 38,83 | 2 | 4 | 4 | DNIQGITKPAIR | 9 | 11,4 |
|  |  |  |  |  |  |  |  |  | ISGLIYEETR |  |  |
|  |  |  |  |  |  |  |  |  | VFLENVVR |  |  |
|  |  |  |  |  |  |  |  |  | DAVTYTEHAR |  |  |
| 0 | C | KAG2432108.1 | hypothetical protein [Chlamydomonas incerta] | 237,66 | 44,67 | 6 | 3 | 3 | TITLEVESSDTIENVK | 8 | 76,8 |
|  |  |  |  |  |  |  |  |  | ESTLHLVLR |  |  |
|  |  |  |  |  |  |  |  |  | TLADYNIQK |  |  |
| 0 | A,B | KAF8059712.1 | hypothetical protein [Scenedesmus sp. PABB004] | 234,91 | 4,58 | 2 | 1 | 4 | GTLDAFGGEFVVPSYR | 8 | 95,8 |
|  |  |  |  |  |  |  |  |  | VPFLFTVK |  |  |
|  |  |  |  |  |  |  |  |  | ITGLWYAQLK |  |  |
|  |  |  |  |  |  |  |  |  | GSAVFSVAK |  |  |
| 0 | A | KAF8061345.1 | UBQ10 [Scenedesmus sp. PABB004] | 179,68 | 44,64 | 6 | 3 | 3 | TITLEVESSDTIENVK | 5 | 51,6 |
|  |  |  |  |  |  |  |  |  | ESTLHLVLR |  |  |
|  |  |  |  |  |  |  |  |  | TLADYNIQK |  |  |
| 0 | B | KAG1665432.1 | hypothetical protein [Chlamydomonas sp. UWO 241] | 144,68 | 32,00 | 61 | 3 | 3 | ISGLIYEETR | 6 | 11,1 |
|  |  |  |  |  |  |  |  |  | DNIQGITKPAIR |  |  |
|  |  |  |  |  |  |  |  |  | DAVTYTEHAR |  |  |
| M | A | KAF6258034.1 | ATP synthase beta chain mitochondrial precursor [Scenedesmus sp. NREL 46B-D3] | 125,27 | 4,70 | 2 | 1 | 2 | DVEGQDVLLFVDNIFR | 3 | 62,0 |
|  |  |  |  |  |  |  |  |  | IGLFGGAGVGK |  |  |
| 0 | B | GIM07828.1 | hypothetical protein, partial [Volvox reticuliferus] | 125,02 | 39,42 | 87 | 2 | 2 | TITLEVESSDTIENVK | 3 | 11,6 |
|  |  |  |  |  |  |  |  |  | ESTLHLVLR |  |  |
| 0 | B | CBN20770.1 | calmodulin, partial [Polytomella sp. Pringsheim 198.80] | 109,48 | 22,00 | 17 | 2 | 2 | EADVDGDGQVNYEEFVK | 3 | 17,0 |
|  |  |  |  |  |  |  |  |  | VFDKDGNGFISAAELR |  |  |
| 0 | C | XP_001691148.1 | histone H4, partial [Chlamydomonas reinhardtii] | 105,52 | 37,29 | 8 | 2 | 2 | ISGLIYEETR | 3 | 6,4 |
|  |  |  |  |  |  |  |  |  | DNIQGITKPAIR |  |  |
| 0 | A | KAF8071269.1 | actin [Scenedesmus sp. PABB004] | 99,12 | 10,08 | 2 | 3 | 3 | AGFAGDDAPR | 5 | 41,7 |
|  |  |  |  |  |  |  |  |  | GYSFTTTAER |  |  |
|  |  |  |  |  |  |  |  |  | VAPEEHPVLLTEAPLNPK |  |  |
| C | A | ABD37899.1 | light-harvesting chlorophyll-a/b binding protein LhcbM2 [Tetradesmus obliquus] | 95,32 | 14,86 | 15 | 3 | 3 | WAmLGALGcITPELLAK | 4 | 26,4 |
|  |  |  |  |  |  |  |  |  | NGVPFGEAVWFK |  |  |
|  |  |  |  |  |  |  |  |  | EIEVIHAR |  |  |
| 0 | C | XP_001699068.1 | actin [Chlamydomonas reinhardtii] | 89,38 | 10,08 | 1 | 3 | 3 | AGFAGDDAPR | 4 | 41,8 |
|  |  |  |  |  |  |  |  |  | GYSFTTTAER |  |  |
|  |  |  |  |  |  |  |  |  | VAPEEHPVLLTEAPLNPK |  |  |
| S | A,B | KAF6261957.1 | hypothetical protein [Scenedesmus sp. NREL 46B-D3] | 87,37 | 0,99 | 3 | 2 | 2 | ALEAQLVADAVAAR | 3 | 229,4 |
|  |  |  |  |  |  |  |  |  | WVTTNIAR |  |  |
| M | A | KAF8073224.1 | GapC [Scenedesmus sp. PABB004] | 79,85 | 1,37 | 3 | 2 | 2 | VGINGFGR | 3 | 145,3 |
|  |  |  |  |  |  |  |  |  | AGImLSPTFVK |  |  |
| 0 | A | BAC20391.1 | ATP synthase beta-subunit, partial [Scenedesmus quadricauda] | 79,82 | 5,85 | 9 | 1 | 2 | IGLFGGAGVGK | 3 | 40,7 |
|  |  |  |  |  |  |  |  |  | TAPAFVDLDTR |  |  |
| 0 | A | KAF6254215.1 | 14-3-3-like protein-related protein [Scenedesmus sp. NREL 46B-D3] | 65,73 | 7,00 | 2 | 2 | 2 | DSTLIMQLLR | 2 | 29,5 |
|  |  |  |  |  |  |  |  |  | NLLSVAYK |  |  |
| 0 | A,B | KAF8071184.1 | enol-1 [Scenedesmus sp. PABB004] | 63,45 | 1,97 | 2 | 2 | 2 | GNPTVEAEVTTFK | 2 | 164,6 |
|  |  |  |  |  |  |  |  |  | AAVPSGASTGIHEAVELR |  |  |
| C | A | KAF6257693.1 | phosphoglycerate kinase precursor [Scenedesmus sp. NREL 46B-D3] | 61,78 | 4,58 | 3 | 2 | 2 | VLPGVAALDEK | 2 | 46,6 |
|  |  |  |  |  |  |  |  |  | ADLNVPLDK |  |  |
| 0 | C | AHB29568.1 | ATP synthase beta-subunit, partial [Chlamydomonas reinhardtii] | 54,10 | 10,09 | 10 | 2 | 2 | IGLFGGAGVGK | 2 | 23,5 |
|  |  |  |  |  |  |  |  |  | TAPAFVDLDTR |  |  |
| 0 | A,B | KAF6261334.1 | hypothetical protein [Scenedesmus sp. NREL 46B-D3] | 199,56 | 1,92 | 1 | 1 | 1 | SQAQcFTQPGYGR | 4 | 69,9 |
| 0 | A,B | KAF6254028.1 | HSP20-like chaperone [Scenedesmus sp. NREL 46B-D3] | 135,97 | 5,11 | 1 | 1 | 1 | VAEGVLTITVPK | 2 | 25,8 |
| 0 | A,B | KAF6260312.1 | hypothetical protein [Scenedesmus sp. NREL 46B-D3] | 128,47 | 4,66 | 1 | 1 | 1 | LPPLPNVDLSK | 4 | 25,5 |
| 0 | A | KAF8071034.1 | zinc finger CCCH domain-containing protein 50 [Scenedesmus sp. PABB004] | 121,16 | 1,03 | 1 | 1 | 1 | AEVLDALR | 4 | 77,8 |
| 0 | B | GAX81993.1 | hypothetical protein [Chlamydomonas eustigma] | 120,82 | 12,07 | 3 | 1 | 1 | SPNSDTYIIFGEAK | 2 | 12,7 |
| S | A,B | KAF6251784.1 | hypothetical protein [Scenedesmus sp. NREL 46B-D3] | 116,68 | 0,70 | 3 | 1 | 1 | GSSDScYIQR | 2 | 148,6 |
| S | A | KAF8072813.1 | EXPA15 [Scenedesmus sp. PABB004] | 116,19 | 1,89 | 1 | 1 | 1 | WGVIAIEWR | 2 | 52,4 |
| 0 | A | ACN59931.1 | translation elongation factor-like protein, partial [Tetradesmus obliquus] | 111,50 | 3,44 | 3 | 1 | 1 | LLFELGGIPER | 2 | 35,4 |
| S | A,B | KAF6264072.1 | hypothetical protein [Scenedesmus sp. NREL 46B-D3] | 109,74 | 1,89 | 1 | 1 | 1 | GTYNVGSNTAGcQK | 2 | 76,3 |
| 0 | B | BAF42664.1 | elongation factor-1 like protein, partial [Pleodorina starrii] | 103,82 | 13,25 | 34 | 1 | 1 | LLFELGGIPER | 2 | 9,4 |
| S | B | XP_013899571.1 | hypothetical protein [Monoraphidium neglectum] | 101,69 | 4,17 | 2 | 1 | 1 | WGVIALEWR | 2 | 24,4 |
| 0 | A | KAF6260750.1 | putative calmodulin [Scenedesmus sp. NREL 46B-D3] | 98,34 | 10,69 | 2 | 1 | 1 | EADVDGDGQVNYEEFVK | 2 | 18,2 |
| 0 | C | XP_001693997.1 | beta tubulin 2 [Chlamydomonas reinhardtii] | 97,02 | 2,26 | 1 | 1 | 1 | FPGQLNADLR | 2 | 49,6 |
| S | A,B | KAF8073058.1 | celI [Scenedesmus sp. PABB004] | 96,66 | 0,86 | 2 | 1 | 1 | dLSGGYYDAGDYLK | 2 | 167,8 |
| 0 | A | KAF6240835.1 | histone-fold-containing protein [Scenedesmus sp. NREL 46B-D3] | 94,02 | 7,26 | 9 | 1 | 1 | AGLQFPVGR | 2 | 13,2 |
| 0 | A,B | KAF8055287.1 | RBCS-2 [Scenedesmus sp. PABB004] | 79,26 | 9,23 | 1 | 1 | 1 | mGAVSSNYFDNR | 1 | 14,9 |
| M | B | GBF92678.1 | cytochrome c oxidase subunit 4, 13 kD [Raphidocelis subcapitata] | 77,78 | 11,52 | 1 | 1 | 1 | IIGVTDPEDDTLVIWGIIR | 1 | 17,8 |
| C | C | P11471.1 | Oxygen-evolving enhancer protein 2, chloroplastic [Chlamydomonas reinhardtii] | 76,74 | 4,49 | 2 | 1 | 1 | FLESVSYLLGK | 2 | 25,9 |
| C | A,B | KAF8064575.1 | hypothetical protein [Scenedesmus sp. PABB004] | 71,91 | 3,34 | 1 | 1 | 1 | IQDAGTEVVEAK | 1 | 37,0 |
| S | A,B | KAF6256986.1 | Six-hairpin glycosidase-like protein [Scenedesmus sp. NREL 46B-D3] | 70,75 | 1,42 | 1 | 1 | 1 | cPAAPYSTVcYTAK | 1 | 105,2 |
| 0 | A | KAF8069453.1 | CHR11 [Scenedesmus sp. PABB004] | 68,43 | 0,37 | 1 | 1 | 1 | IAADIEGK | 2 | 229,5 |
| 0 | C | ACN59930.1 | translation elongation factor-like protein, partial [Chlamydomonas reinhardtii] | 67,00 | 3,44 | 3 | 1 | 1 | LLFELGGIPER | 1 | 35,3 |
| 0 | B | CAD8307465.1 | unnamed protein product, partial [Chlamydomonas euryale] | 64,82 | 10,89 | 365 | 1 | 1 | IGLFGGAGVGK | 2 | 10,8 |
| 0 | B | CAD8675177.1 | unnamed protein product, partial [Chlamydomonas leiostraca] | 62,97 | 4,26 | 39 | 1 | 1 | FPGQLNADLR | 1 | 26,8 |
| 0 | A | KAF6258365.1 | beta-tubulin [Scenedesmus sp. NREL 46B-D3] | 62,97 | 2,26 | 2 | 1 | 1 | FPGQLNADLR | 1 | 49,6 |
| 0 | A | ADV77211.1 | histone H3, partial [Tetradesmus obliquus] | 61,56 | 5,93 | 7 | 1 | 1 | STELLIR | 1 | 13,2 |
| S | A,B | KAF6264724.1 | hypothetical protein [Scenedesmus sp. NREL 46B-D3] | 59,84 | 3,57 | 1 | 1 | 1 | QVYPLSSFK | 2 | 28,4 |
| 0 | B | GFH18485.1 | phosphoglycerate kinase [Haematococcus lacustris] | 57,56 | 7,05 | 18 | 1 | 1 | VLPGVAALDEK | 1 | 15,7 |
| C | A | KAF8057635.1 | hypothetical protein [Scenedesmus sp. PABB004] | 57,55 | 0,91 | 1 | 1 | 1 | SGFVPYAGEGFAILLPSK | 2 | 195,3 |
| 0 | A | YP_009450196.1 | photosystem I subunit VII [Pectinodesmus pectinatus] | 57,10 | 11,11 | 2 | 1 | 1 | VYLSSETTR | 2 | 8,9 |
| 0 | C | XP_001697137.1 | predicted protein, partial [Chlamydomonas reinhardtii] | 56,70 | 0,54 | 2 | 1 | 1 | lSEVLHAR | 2 | 160,0 |
| 0 | C | XP_001696218.1 | histone H2A, partial [Chlamydomonas reinhardtii] | 55,73 | 18,00 | 10 | 1 | 1 | AGLQFPVGR | 1 | 5,2 |
| 0 | A,B | KAF6264795.1 | glycoside hydrolase, partial [Scenedesmus sp. NREL 46B-D3] | 54,28 | 3,02 | 5 | 1 | 1 | DVTGGWYDAGDNVK | 1 | 100,7 |
| 0 | C | XP_001700833.1 | R1 protein, alpha-glucan water dikinase [Chlamydomonas reinhardtii] | 52,61 | 0,50 | 2 | 1 | 1 | VLDELWR | 1 | 154,2 |
| S | A,B | KAF6266133.1 | RlpA-like double-psi beta-barrel-protein domain-containing protein-containing protein [Scenedesmus sp. NREL 46B-D3] | 52,41 | 5,91 | 1 | 1 | 1 | ATFYGVDAWSIHK | 1 | 24,7 |
| 0 | C | XP_001703200.1 | glyceraldehyde 3-phosphate dehydrogenase, minor splicing variant [Chlamydomonas reinhardtii] | 50,46 | 3,46 | 3 | 1 | 1 | AGImLSPTFVK | 2 | 34,1 |
| 0 | A | KAF8060334.1 | AAK6 [Scenedesmus sp. PABB004] | 49,42 | 0,31 | 1 | 1 | 1 | LVVDIGPLIGK | 1 | 355,2 |
| 0 | B | 7D0J_P | unnamed protein product [Chlamydomonas reinhardtii] | 48,88 | 7,76 | 36 | 1 | 1 | WAmLGALGcITPELLAK | 1 | 23,7 |
| 0 | B | GIM13818.1 | hypothetical protein, partial [Volvox reticuliferus] | 48,10 | 1,41 | 5 | 1 | 1 | YEEAELLYR | 1 | 69,2 |
| 0 | A | KAF8071022.1 | hypothetical protein [Scenedesmus sp. PABB004] | 48,00 | 2,05 | 2 | 1 | 1 | YLYSQTAK | 1 | 43,3 |
| 0 | B | CAD8302002.1 | unnamed protein product, partial [Chlamydomonas euryale] | 47,21 | 4,10 | 23 | 1 | 1 | DSTLIMQLLR | 1 | 27,7 |
| 0 | C | XP_001702812.1 | 14-3-3 protein [Chlamydomonas reinhardtii] | 47,21 | 3,86 | 1 | 1 | 1 | DSTLIMQLLR | 1 | 29,5 |
| C | C | P12853.1 | Oxygen-evolving enhancer protein 1, chloroplastic [Chlamydomonas reinhardtii] | 45,75 | 3,09 | 2 | 1 | 1 | GSAVFSVAK | 2 | 30,5 |
| 0 | B | GBF88582.1 | chlorophyll a-b binding protein, chloroplastic [Raphidocelis subcapitata] | 45,48 | 3,85 | 3 | 1 | 1 | ALFLPGGLLDR | 1 | 29,7 |
| 0 | A | KAF6257400.1 | minor chlorophyll a/b-binding protein of photosystem II [Scenedesmus sp. NREL 46B-D3] | 45,48 | 3,81 | 2 | 1 | 1 | ALFLPGGLLDR | 1 | 30,6 |
| 0 | A | KAF8072472.1 | hypothetical protein [Scenedesmus sp. PABB004] | 45,38 | 0,28 | 1 | 1 | 1 | AGALLEAR | 1 | 284,9 |
| T | B | GBF97368.1 | hypothetical protein [Raphidocelis subcapitata] | 44,88 | 7,17 | 3 | 1 | 1 | SGFVPYAGEGFAILLPSK | 1 | 26,4 |
| M | A,B | KAF8056367.1 | hypothetical protein [Scenedesmus sp. PABB004] | 44,41 | 10,43 | 1 | 1 | 1 | FGTEDNPIQVPALLSER | 1 | 17,2 |
| 0 | B | XP_013890366.1 | ADP,ATP carrier protein [Monoraphidium neglectum] | 42,74 | 5,08 | 24 | 1 | 1 | YFPTQALNFAFK | 1 | 26,1 |
| 0 | A | KAF6257963.1 | mitochondrial ADP/ATP translocator [Scenedesmus sp. NREL 46B-D3] | 42,74 | 3,85 | 2 | 1 | 1 | YFPTQALNFAFK | 1 | 34,1 |
| M | A | KAF6254630.1 | translation elongation factor, mitochondrial [Scenedesmus sp. NREL 46B-D3] | 42,45 | 2,04 | 2 | 1 | 1 | TTLTAAITK | 2 | 47,7 |
| 0 | A | KAF6248398.1 | histone H2B [Scenedesmus sp. NREL 46B-D3] | 42,39 | 7,44 | 9 | 1 | 1 | LILPGELAK | 1 | 13,2 |
| T | A | KAF6250768.1 | chloroplast photosystem I subunit N [Scenedesmus sp. NREL 46B-D3] | 41,99 | 6,94 | 2 | 1 | 1 | LATSSANVAR | 1 | 15,3 |
| 0 | C | XP_001696344.1 | mitochondrial translation factor Tu, partial [Chlamydomonas reinhardtii] | 41,31 | 2,28 | 2 | 1 | 1 | TTLTAAITK | 2 | 42,8 |
| C | A | KAF6255847.1 | fructose-bisphosphate aldolase 1, chloroplastic [Scenedesmus sp. NREL 46B-D3] | 40,84 | 3,45 | 2 | 1 | 1 | LESIGLENTEDNR | 1 | 41,2 |
| T | A | KAF6260931.1 | hypothetical protein [Scenedesmus sp. NREL 46B-D3] | 40,52 | 5,09 | 2 | 1 | 1 | QLDSELLPVQK | 1 | 23,0 |
| 0 | A | KAF8060314.1 | RTNLB4 [Scenedesmus sp. PABB004] | 40,51 | 1,92 | 1 | 1 | 1 | gANVALSLLGR | 2 | 59,3 |
| 0 | A | KAF6263992.1 | hypothetical protein [Scenedesmus sp. NREL 46B-D3] | 40,00 | 0,67 | 1 | 1 | 1 | lSEQLAAAR | 1 | 149,2 |
| C | B | ABA01126.1 | glyceraldehyde-3-phosphate dehydrogenase, partial [Chlamydomonas incerta] | 39,53 | 5,64 | 17 | 1 | 1 | AGImLSPTFVK | 1 | 20,7 |
| 0 | B | AAR08365.1 | ATPase alpha subunit, partial [Dunaliella salina] | 38,29 | 9,63 | 111 | 1 | 1 | EAYPGDVFYIHSR | 2 | 14,7 |
| 0 | A | AOY36020.1 | ATP synthase CF1 alpha subunit [Hariotina sp. MMOGRB0030F] | 38,29 | 2,56 | 7 | 1 | 1 | EAYPGDVFYLHSR | 2 | 54,4 |
| S | A,B | KAF6251358.1 | Calreticulin family-domain-containing protein [Scenedesmus sp. NREL 46B-D3] | 38,21 | 3,09 | 1 | 1 | 1 | AETDQLTHVYTLR | 1 | 47,9 |
| S | A | KAF8060350.1 | hypothetical protein [Scenedesmus sp. PABB004] | 37,99 | 0,98 | 1 | 1 | 1 | LYQFTQGR | 2 | 80,3 |
| 0 | B | GFH14075.1 | actin beta/gamma 1, partial [Haematococcus lacustris] | 37,64 | 18,56 | 27 | 1 | 1 | VAPEEHPVLLTEAPLNPK | 2 | 11,2 |
| S | A | KAF6264590.1 | heat shock protein 70 family [Scenedesmus sp. NREL 46B-D3] | 34,50 | 1,77 | 6 | 1 | 1 | VEIIANDQGNR | 1 | 68,1 |
| 0 | C | P25840.2 | Heat shock 70 kDa protein [Chlamydomonas reinhardtii] | 34,50 | 1,69 | 5 | 1 | 1 | VEIIANDQGNR | 1 | 71,0 |
| 0 | A | QEM20955.1 | ribulose-1,5-bisphosphate carboxylase/oxygenase large subunit, partial [Coelastrella oocystiformis] | 33,22 | 7,28 | 129 | 1 | 1 | EVTLGFVDLmR | 1 | 16,7 |
| 0 | C | PNW80233.1 | hypothetical protein [Chlamydomonas reinhardtii] | 32,20 | 0,54 | 1 | 1 | 1 | SGGGGGVEVSSPSR | 1 | 250,8 |
| 0 | A | KAF6254005.1 | hypothetical protein [Scenedesmus sp. NREL 46B-D3] | 29,65 | 0,62 | 1 | 1 | 1 | SGQLEVYSR | 1 | 153,7 |
| S | A | KAF8064679.1 | pitC [Scenedesmus sp. PABB004] | 29,63 | 0,81 | 1 | 1 | 1 | FVPcGGPNPDYK | 1 | 158,9 |
| M | A | KAF6259622.1 | thioredoxin-like protein [Scenedesmus sp. NREL 46B-D3] | 27,59 | 5,53 | 1 | 1 | 1 | ALGVELDLADK | 1 | 20,9 |
| 0 | C | 1206346A | calmodulin [Chlamydomonas reinhardtii] | 27,14 | 9,88 | 2 | 1 | 1 | VFDKDGNGFISAAELR | 1 | 18,2 |
| 0 | A | KAF8058425.1 | hypothetical protein [Scenedesmus sp. PABB004] | 24,17 | 0,61 | 1 | 1 | 1 | WGWIGDTR | 1 | 136,1 |
| S | A | KAF8059426.1 | BGLU42 [Scenedesmus sp. PABB004] | 22,96 | 0,76 | 1 | 1 | 1 | LSIALDGK | 1 | 109,9 |
| 0 | C | CAA42840.1 | adenosinetriphosphatase, partial [Chlamydomonas reinhardtii] | 22,86 | 2,86 | 3 | 1 | 1 | EAYPGDVFYLHSR | 1 | 48,7 |
